# Spinal anaesthesia in a restless eclamptic with undiagnosed second twin: case report

**DOI:** 10.11604/pamj.2015.20.111.5012

**Published:** 2015-02-06

**Authors:** Jide Michael Afolayan, Babatunde Ajayi Olofinbiyi, Oluwadare Martins Ipinnimo

**Affiliations:** 1 Department of Anaesthesia, Ekiti State University Teaching Hospital, Ado-Ekiti, Nigeria; 2 Department of Obstetric and Gynaecology, Ekiti State University Teaching Hospital, Ado-Ekiti, Nigeria

**Keywords:** Eclampsia, caesarean section, general anaesthesia, spinal anaesthesia

## Abstract

The use of sub-arachnoid block in a restless eclamptic is not very common. Studies have demonstrated some benefits of sub-arachnoid block over general anaesthesia in stable eclamptic but its role in the management of unstable eclampsia has not been established. Reported below is an eclamptic parturient who was restless despite magnesium sulphate regimen and possesed features suggestive of difficult airway who had uneventful subarachnoid- block for caesarean section.

## Introduction

Spinal anaesthesia in stable eclampsia is no longer new [1–6]. Eclampsia is said to be unstable if there are associated complications such as thrombocytopenia, raised intra cranial pressure, uncontrollable convulsions, restlessness, fetal distress, unconsciousness (GCS < 9), respiratory failure and haemorrhage [4, 5]. Studies have demonstrated some benefits of sub-arachnoid block over general anaesthesia in stable eclamptic [1, 2, 4–6]. The use of sub-arachnoid block in a restless eclamptic has not been documented. James et al administered spinal anaesthesia to unstable eclamptic patients in 1912 without recording any mortality having ealier on had serial mortalities with general anaesthesia in patients with eclampsia [1]. Paramore actually administered spinal anaesthesia to an unstable eclamptic in 1930 with the aim of using it to abort convulsions [2]. Faced with anaesthetic dilemma, Nafiu et al offered spinal anaesthesia to an unstable eclamptic patient with mild thrombocytopenia and an impossible airway without history of spinal haematoma [4]. We present anaesthetic management in an unstable eclamptic parturient who was restless despite magnesium sulphate regimen and possesed features suggestive of difficult airway.

## Patient and observation

Mrs.G. B was a 23 year old Gravida 1 para 0 trader who resides at Okela street Ado-Ekiti, Nigeria. She was transferred from a comprehensive health centre to the Ekiti State University Teaching Hospital in Ado-Ekiti, Ekiti State, Southwest Nigeria, on the 3^rd^ of April, 2014 at 11.00hrs. She presented on account of convulsion of several episodes at the referring comprehensive Health Centre before presentation at the hospital. Convulsions were said to be tonic-clonic in nature with associated drooling of saliva. There was no fecal or urinary incontinence. No history of post ictal sleep. She had regular menstrual cycles of 5 days in a 30 days cycle prior to conception. The first day of Her last menstrual period could not be ascertained. She had all her antenatal follow up at the referring comperensive health centre. However, the result of the first ultrasound scanning done was not available for perusal. Since admission, she had diazepam but subsequently had another episode of convulsion. She was later given magnesium sulphate which abated further convulsion. There was no previous history of convulsion before the index pregnancy. No prior history of head trauma. No family or previous history of epilepsy. Nil previous anaesthetic exposure nor blood transfusion. No history of drug allergy. Nil intercurrent medical ailment predating pregnancy. On examination she was drowsy and restless. Glasglow coma scale could not be assesed. She was not pale and anicteric, midly dehydrated. Her pulse rate was 120 bpm, Blood pressure was 160/100 mmHg, respiratory rate was 38 cycles per minute. Her lung field was clear clinically. Abdominal examination reveals a uniformly enlarged uterus with symphysio-fundal height of 32 cm compactible with a gestational age of 34 weeks. Longitudinal lie, cephalic presenting fetus. She was having two uterine contractions in 10 minutes each lasting for 30 seconds. Fetal heart sound was 140 beats/minute. She had normal vulva, a posteriorly located closed cervix measuring 2cm long and 80% effaced. There was no vaginal bleeding.

After about 4 hours of stabilisation, she was later scheduled for emergency caesarean section. She was held in place on the operating table because she was restless. Monitors were attached. Intravenous access was achieved with 18-G cannula. Owing to the possibility of difficult airway, plan was to administered sub-arachnoid block. Intravenous midazolam 4mg in aliquots of 2 mg was given to manage her restlessness. Thereafter she was calm and had a preload of 750 mls of normal saline. The patient received ranitidine 50mg and 10mg metoclopramide intravenously 30 minutes prior to surgery. Following the application of routine monitoring, non-invasive monitoring was commenced and documented including non-invasive blood pressure (NIBP), oxygen saturation (SPO_2_), pulse (PR), systolic blood pressure (SBP), diastolic blood pressure (DBP). Induction of spinal anaesthesia was achieved with patient held by two assistants in sitting position. Her legs were completely kept straight on the operating table, unlike the conventional method where legs were hanging from the edge of the operating table with the support of a stool under her feet. She was assisted in bending her neck forward and arching out her back maximally. Under aseptic condition, the spinal needle was introduced into the subarachnoid space. After withdrawing the stylet from the spinal needle, appearance of the free flow of cerebrospinal fluid in the hub of the needle indicated a successful placement. She received 2.2ml of 0.5% hyperbaric bupivacaine plus 10 mg pethidine over 15s intrathecally in the L3-4 intervertebral space with a 25 G Quincke's spinal needle. The patient was immediately put in supine position with a 15° left lateral tilt using wedge under the right hip. Sensory bock height was assessed using loss of sensation to gentle pin prick test. A sensory block height of T6 was the minimum desired level of block for the commencement of the caesarean section. The following parameters: pulse rate, systolic blood pressure, diastolic blood pressure, and oxygen saturation were recorded. Following the delivery of the first twin, the mother was inadvertently given 10 units of oxytocin intravenously be the delivery of the second twin, then she had infusion of 40 units of oxytocin in 500 mls normal saline to run for 4 hours. One minute Apgar scores for first and second twins were 6 and 7 respectively. Postoperatively, she was transferred to the ward where she became conscious within 24 hours following anaesthesia. She was discharged home alongside her two neonates having spent seven days in the ward.

## Discussion

The patient presented in this case report was having persistent restlessness and drowsiness despite management with magnesium sulphate for 4 hours prior to spinal anaesthesia. It has also been documented by Paramore et al that spinal anaesthesia could actually be used to prevent further restlessness and convulsions in eclampsia [2]. General anaesthesia would have been a technique of choice in this patient with unstable eclampsia, but the presence of features suggestive of difficult airway did not favour it. In the face of difficult intubation, the attendant hypoxia (desaturation), aspiration pneumonitist, aggravated laryngeal oedema, pressor response to laryngoscopic and intubation can be exaggerated in this eclamptic parturient [5, 6]. Although Mallampati assessment could not be carried out, the patient has short neck, large and swollen tongue and more swollen face due to significant tissue edema. It is recommended that regional anaesthesia as the best possible choice in most cases of anticipated difficult airway. Spinal anaesthesia supports haemodynamic stability, avoids multiple drug interractions (especially, unwarranted potentiating of non-depolarizing muscle relaxant by magnesium sulphate [7]). The temporary difficulty in positioning her for induction of spinal anaesthesia was alleviated with the help of two assistants who positioned her on the operating table. Because the patient was drowsy, two assistants put her in sitting position on the operating table. We also did not have difficult or failed spinal anaesthesia. This finding was in support of findings of Singh et al and Basu et al who had no difficult or failed spinal anaesthesia amongst the eclamptic patients who had spinal aneasthesia [5, 6]. The procedure which was performed by a consultant anaesthetist was not associated with any form of difficulty in passing spinal needle nor spinal haematoma. The platelet count in our patient was normal. Yuen et al observed spinal haematoma in a patient with a twin pregnancy required a Caesarean section for severe pre-eclampsia [8]. She presented with platelet count of 71 × 10^9^ cells/L. She had spinal haematoma following epidural anaesthesia. Razzaque et al. demonstrated safety of spinal anaesthesia in stable eclampsia and concluded that spinal anaesthesia is safer than general anaesthesia for Lower Segment Caesarean Section in eclamptics [9].

We used 26 G spinal needle for this present patient. Even in the face of thrombocytopenia, Nafiu et al did not record any spinal haematoma after a successful spinal anaesthesia in an unstable eclamptic patient with thrombocytopenia [4]. For most centres, the lowest acceptable platelet count for subarachnoid block is still a subject of controversy. However some authors peg the minimal allowable count at 100 × 10^9^cells/L [4–6]. Basu et al reported that 11 out of 30 neonates in eclamptics with general anesthesia group had resuscitatation with Ambubag-mask ventilation compared to 2 in spinal anaesthesia [6]. Dasqupta et al Compared neonatal outcome in women with severe pre-eclampsia undergoing Caesarean section under spinal or general anesthesia and found that neonates in mothers with spinal anaesthesia had better Apgar scores [10]. This was in accordance with our study where the two neonates had 1 min Apgar score of 6 and 7. Basu et al found that parturients with general anaesthesia was associated with prolonged hospital stay. This corroborated our finding where the patient was discharged after 7 days of admission. The case presented was one of the few selected cases of unstable eclamptic parturients that can benefit from spinal anaesthesia for caesearen section. Although spinal anaesthesia has been confirmed to be useful in stable eclamptcs, its use in unstable eclamptics should be individualized. Apart from patients with thrombocytopenia and raised intra cranial pressure, spinal anaesthesia may be favoured in some selected cases.

## Conclusion

The case reported highlights the use of an uneventful spinal anaesthesia for caesarean section in eclamptic with unaborted restlessness and unconsciousness. The case presented was one of the few selected cases of unstable eclamptic parturients that can benefit from spinal anaesthesia for caesearen section.
